# Na_5_(NH_4_)Mn_3_[B_9_P_6_O_33_(OH)_3_]·1.5H_2_O

**DOI:** 10.1107/S1600536808037884

**Published:** 2008-11-22

**Authors:** Zhi-Sheng Lin, Ya-Xi Huang, Yurii Prots, Jing-Tai Zhao, Rüdiger Kniep

**Affiliations:** a State Key Laboratory of High Performance Ceramics and Superfine Microstructures, Shanghai Institute of Ceramics, Chinese Academy of Sciences, Shanghai 200050, People’s Republic of China; b Max-Planck-Institut für Chemische Physik fester Stoffe, Nöthnitzer Strasse 40, 01187 Dresden, Germany; c Graduate School of Chinese Academy of Sciences, Beijing, People’s Republic of China; d Department of Materials Science and Engineering, College of Materials, Xiamen University, Xiamen 361005, People’s Republic of China

## Abstract

The overall hexa­gonal framework of the title compound, penta­sodium ammonium trimanganese(II) borophosphate sesquihydrate, consists of tube-like borophosphate anions, _∞_^1^{[B_3_P_2_O_11_(OH)]^4−^}, made up of corner-sharing PO_4_ and BO_4_ tetra­hedra and BO_2_(OH) triangles, forming ten-membered ring windows. The tubes are inter­connected *via* distorted MnO_6_ octa­hedra, establishing a three-dimensional open-framework structure with two different types of ring-channels (12- and six-membered) that run along [001]. The 12-membered ring channels are occupied by NH_4_^+^ ions and water mol­ecules. The ten-membered ring windows in the walls of the tubes are occupied by Na^+^ ions. The remaining Na^+^ ions and the water mol­ecules, one of which is half-occupied, reside within the six-membered ring channels. The structural setup is consolidated by an O—H⋯O hydrogen bond between the OH group and an opposite O atom of the framework. Donor–acceptor distances ranging from 2.80 to 3.35 Å between the ammonium N atom, water O atoms and framework O atoms indicate further hydrogen-bonding inter­actions.

## Related literature

Reviews on the preparation, crystal chemistry and applications of borophosphates are given in Kniep *et al.* (1998 ▶) and Ewald *et al.* (2007 ▶). For isostructural compounds, see Yang, Li *et al.* (2006 ▶) for Na_2_Mn[B_3_P_2_O_11_(OH)]·0.67H_2_O; Yang, Yu *et al.* (2006 ▶) for Na_5_(H_3_O)Mn_3_[B_9_P_6_O_33_(OH)_3_]·2H_2_O; Liu *et al.* (2006 ▶) for Na_6_Cu_3_[B_9_P_6_O_33_(OH)_3_]·2H_2_O.

## Experimental

### 

#### Crystal data

Na_5_(NH_4_)Mn_3_[B_9_P_6_O_33_(OH)_3_]·1.5(H_2_O)
*M* *_r_* = 2373.94
Hexagonal, 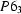

*a* = 11.9331 (2) Å
*c* = 12.1290 (4) Å
*V* = 1495.76 (6) Å^3^
*Z* = 1
Mo *K*α radiation
μ = 1.79 mm^−1^
*T* = 295 K
0.08 × 0.04 × 0.04 mm

#### Data collection

Rigaku Mercury AFC7 CCD diffractometer
Absorption correction: multi-scan (Blessing, 1995 ▶) *T*_min_ = 0.779, *T*_max_ = 0.931
12243 measured reflections
2891 independent reflections
2784 reflections with *I* > 2σ(*I*)
*R*_int_ = 0.030

#### Refinement

*R*[*F* ^2^ > 2σ(*F* ^2^)] = 0.040
*wR*(*F* ^2^) = 0.099
*S* = 1.12
2891 reflections
191 parameters
1 restraint
H atoms treated by a mixture of independent and constrained refinement
Δρ_max_ = 0.66 e Å^−3^
Δρ_min_ = −0.81 e Å^−3^
Absolute structure: Flack (1983 ▶), 1374 Friedel pairs
Flack parameter: 0.43 (3)

### 

Data collection: *CrystalClear* (Rigaku/MSC, 2005 ▶); cell refinement: *CrystalClear*; data reduction: *CrystalClear*; program(s) used to solve structure: *SHELXS97* (Sheldrick, 2008 ▶); program(s) used to refine structure: *SHELXL97* (Sheldrick, 2008 ▶); molecular graphics: *DIAMOND* (Brandenburg, 2004 ▶); software used to prepare material for publication: *SHELXL97*.

## Supplementary Material

Crystal structure: contains datablocks I, ZSL_079. DOI: 10.1107/S1600536808037884/wm2204sup1.cif

Structure factors: contains datablocks I. DOI: 10.1107/S1600536808037884/wm2204Isup2.hkl

Additional supplementary materials:  crystallographic information; 3D view; checkCIF report

## Figures and Tables

**Table 1:** Hydrogen-bond geometry (Å, °)

*D*—H⋯*A*	*D*—H	H⋯*A*	*D*⋯*A*	*D*—H⋯*A*
O12—H1⋯O4^i^	0.86 (7)	2.11 (7)	2.959 (3)	167 (3)
O13⋯O10^ii^	–	–	2.8035 (11)	–
O13⋯N^iii^	–	–	3.077 (16)	–
O14⋯O8^iv^	–	–	3.284 (5)	–
O14⋯O6^iv^	–	–	3.333 (3)	–
N⋯O13^v^	–	–	2.988 (16)	–
N⋯O3^vi^	–	–	2.991 (3)	–
N⋯O7^v^	–	–	3.047 (3)	–

Symmetry codes: (i) 
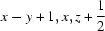
; (ii) 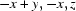; (iii) 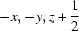; (iv) 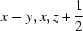; (v) 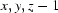; (vi) 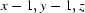.

**Fig. 1.**
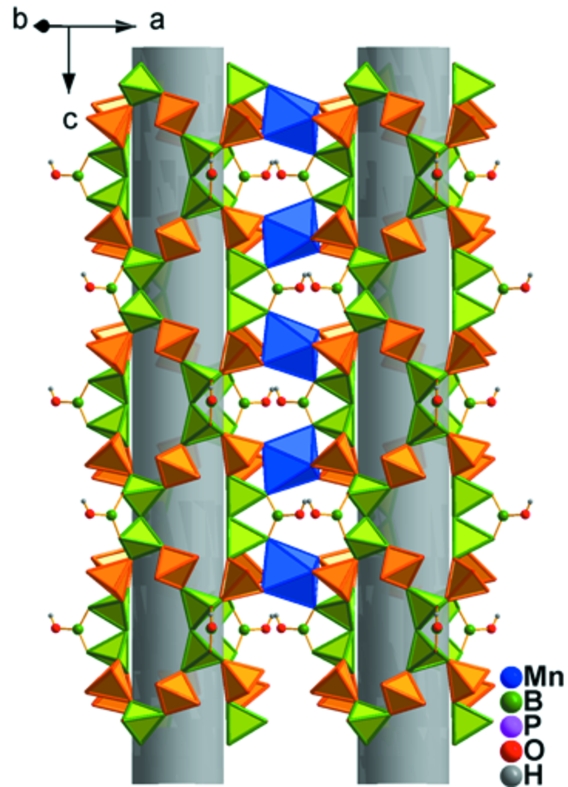
Borophosphate tubes in the crystal structure of Na5(NH4)Mn3[B9P6O33(OH)3].1.5H2O interconnected by MnO6 coordination octahedra.

**Fig. 2.**
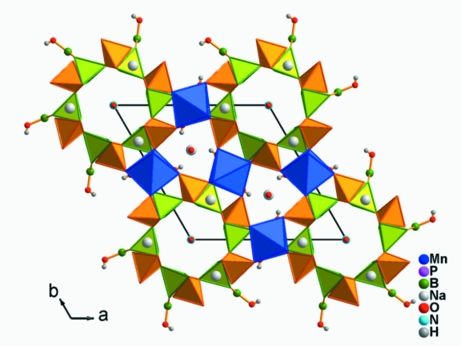
The overall framework of Na5(NH4)Mn3[B9P6O33(OH)3].1.5H2O viewed along [001], showing the resulting channel-system.

## supplementary crystallographic information

### Comment

In the past several years, borophosphates have attracted extensive attention due to their rich structural chemistry and potential applications as catalysts (Kniep *et al.*, 1998; Ewald *et al.*, 2007). Although a large variety of borophosphate anions has been reported, tube-like borophosphate anions are particularly rare (Liu *et al.*, 2006; Yang *et al.*, 2006*a*; Yang *et al.*, 2006*b*). Up to now, only two manganese compounds containing borophosphate tubes, *viz.* Na_2_Mn[B_3_P_2_O_11_(OH)]^.^0.67H_2_O (Yang *et al.*, 2006*a*) and Na_5_(H_3_O)Mn_3_[B_9_P_6_O_33_(OH)_3_]^.^2H_2_O (Yang *et al.*, 2006*b*) are listed in the literature. Here, we report on an ammonium substituted sodium manganese borophosphate, Na_5_(NH_4_)Mn_3_[B_9_P_6_O_33_(OH)_3_]^.^1.5H_2_O.

The crystal structure of the title compound comprises tube-like borophosphate anions, _∞_^1^{[B_3_P_2_O_11_(OH)]^4-^}, which are built from 12-membered rings of alternating BO_4_ and PO_4_ tetrahedra, further interlinked by sharing common O-corners of neighbouring rings, and loop-branched by BO_2_(OH) triangles resulting in 10-membered ring windows on the walls of the tubes (Fig. 1). The manganese atoms are in a distorted octahedral coordination, surrounded by four oxygen atoms from phosphate tetrahedra (O1, O2, O5, O6) and two oxygen atoms from borate tetrahedra (O10, O11). The Mn-coordination octahedra interconnect the neighboring borophosphate tubes to form a three-dimensional framework with two different types of channels (Fig. 2), namely 6- and 12-membered ring channels. The 12-membered ring channels are occupied by NH_4_^+^ ions and water molecules; the 10-membered ring windows in the walls of the tubes are occupied by Na^+^ ions. The remaining Na^+^ ions and water molecules reside in the 6-membered ring channels. The structural setup is consolidated by an O—H···O hydrogen bond between the OH group and an opposite O atom of the framework. Donor-acceptor distances ranging from 2.8 to 3.35 Å between the ammonium N atom, water O atoms and framework O atoms indicate further hydrogen bonding interactions, but the corresponding H atoms were not located.

### Experimental

Transparent, colourless single crystals of the title compound were synthesized hydrothermally from a mixture of H_3_BO_3_ (32.2 mmol), Mn(CH_3_COO)_2_^.^4H_2_O (3 mmol), (NH_4_)_2_HPO_4_ (6.4 mmol), NaF (5 mmol), and water (133.4 mmol). The educt mixture was transferred into a Teflon-lined stainless steel autoclave (internal volume 25 ml) and kept at 513 K for five days. The autoclave was cooled down to ambient temperature by removing out of the oven. The reaction products were washed with hot distilled water (333 K) until the boric acid was completely removed. Finally, the solids were dried in air at 333 K. Hexagonal prismatic crystals were selected for single-crystal diffraction. The NH_4_^+^ content was determined by elemental analysis and confirmed by IR spectroscopy.

### Refinement

The measured crystal was racemically twinned with an approximate twin fraction of 2:3. The hydrogen position bonded to O12 was found in a difference Fourier map and was refined freely. The hydrogen positions of the ammonium N atom and of the uncoordinated water atoms at O13 and O14 were not localized. The occupancy of O13 was refined to 0.50 (2). In the last refinement cycle this value was fixed to 0.50.

### Crystal data

**Table:**

Na_5_(NH_4_)Mn_3_[B_9_P_6_O_33_(OH)_3_]·1.5(H_2_O)	*Z* = 1
*M**_r_* = 2373.94	*F*_000_ = 1164
Hexagonal, *P*6_3_	*D*_x_ = 2.635 Mg m^−^^3^
Hall symbol: P 6c	Mo *K*α radiation λ = 0.71073 Å
*a* = 11.9331 (2) Å	Cell parameters from 7346 reflections
*b* = 11.9331 (2) Å	θ = 2.0–33.6º
*c* = 12.1290 (4) Å	µ = 1.79 mm^−^^1^
α = 90º	*T* = 295 K
β = 90º	Prism, colourless
γ = 120º	0.08 × 0.04 × 0.04 mm
*V* = 1495.76 (6) Å^3^	

### Data collection

**Table:**

Rigaku Mercury AFC7 CCD diffractometer	2891 independent reflections
Radiation source: fine-focus sealed tube	2784 reflections with *I* > 2σ(*I*)
Monochromator: graphite	*R*_int_ = 0.030
*T* = 295 K	θ_max_ = 30.0º
ω–scans	θ_min_ = 2.0º
Absorption correction: multi-scan(Blessing, 1995)	*h* = −16→12
*T*_min_ = 0.779, *T*_max_ = 0.931	*k* = −16→16
12243 measured reflections	*l* = −16→16

### Refinement

**Table:**

Refinement on *F*^2^	Hydrogen site location: difference Fourier map
Least-squares matrix: full	H atoms treated by a mixture of independent and constrained refinement
*R*[*F*^2^ > 2σ(*F*^2^)] = 0.040	*w* = 1/[σ^2^(*F*_o_^2^) + (0.0419*P*)^2^ + 2.5644*P*] where *P* = (*F*_o_^2^ + 2*F*_c_^2^)/3
*wR*(*F*^2^) = 0.099	(Δ/σ)_max_ < 0.001
*S* = 1.12	Δρ_max_ = 0.66 e Å^−^^3^
2891 reflections	Δρ_min_ = −0.81 e Å^−^^3^
191 parameters	Extinction correction: none
1 restraint	Absolute structure: Flack (1983), 1374 Friedel pairs
Primary atom site location: structure-invariant direct methods	Flack parameter: 0.43 (3)
Secondary atom site location: difference Fourier map	

### Special details

**Table:**

Geometry. All e.s.d.'s (except the e.s.d. in the dihedral angle between two l.s. planes) are estimated using the full covariance matrix. The cell e.s.d.'s are taken into account individually in the estimation of e.s.d.'s in distances, angles and torsion angles; correlations between e.s.d.'s in cell parameters are only used when they are defined by crystal symmetry. An approximate (isotropic) treatment of cell e.s.d.'s is used for estimating e.s.d.'s involving l.s. planes.
Refinement. Refinement of *F*^2^ against ALL reflections. The weighted *R*-factor *wR* and goodness of fit *S* are based on *F*^2^, conventional *R*-factors *R* are based on *F*, with *F* set to zero for negative *F*^2^. The threshold expression of *F*^2^ > σ(*F*^2^) is used only for calculating *R*-factors(gt) *etc*. and is not relevant to the choice of reflections for refinement. *R*-factors based on *F*^2^ are statistically about twice as large as those based on *F*, and *R*- factors based on ALL data will be even larger.

### Fractional atomic coordinates and isotropic or equivalent isotropic displacement parameters (Å^2^)

**Table:**

	*x*	*y*	*z*	*U*_iso_*/*U*_eq_	Occ. (<1)
Mn2	0.50444 (6)	0.50073 (7)	0.04544 (11)	0.01330 (12)	
P1	0.62139 (9)	0.81051 (9)	0.00965 (7)	0.0111 (2)	
P2	0.37924 (9)	0.19394 (10)	0.08857 (7)	0.0120 (2)	
B1	0.2928 (4)	0.2502 (4)	0.6961 (4)	0.0118 (7)	
B2	0.2992 (4)	0.2504 (5)	0.8974 (4)	0.0151 (8)	
B3	0.4938 (3)	0.4004 (3)	0.7921 (5)	0.0159 (6)	
O1	0.5750 (3)	0.6785 (3)	0.9591 (3)	0.0169 (6)	
O2	0.7011 (3)	0.9084 (3)	0.9177 (2)	0.0162 (6)	
O3	0.7199 (3)	0.8385 (3)	0.1043 (2)	0.0150 (6)	
O4	0.5126 (3)	0.8296 (3)	0.0515 (3)	0.0177 (5)	
O5	0.2925 (3)	0.0953 (3)	0.1789 (2)	0.0152 (5)	
O6	0.4181 (3)	0.3245 (3)	0.1360 (3)	0.0179 (6)	
O7	0.2859 (3)	0.1639 (3)	0.9886 (3)	0.0182 (6)	
O8	0.6857 (3)	0.5090 (3)	0.0545 (3)	0.0175 (6)	
O9	0.5731 (3)	0.6364 (3)	0.1961 (3)	0.0147 (5)	
O10	0.26579 (18)	0.17981 (18)	0.7974 (3)	0.0132 (3)	
O11	0.4355 (3)	0.3637 (3)	0.8933 (3)	0.0157 (6)	
O12	0.6273 (2)	0.4785 (2)	0.7907 (3)	0.0235 (5)	
H1	0.648 (7)	0.498 (7)	0.723 (6)	0.07 (2)*	
Na1	0.71486 (14)	0.73129 (14)	0.8014 (2)	0.0273 (3)	
Na2	0.3333	0.6667	0.9533 (3)	0.0251 (6)	
N	0.0000	0.0000	0.0452 (12)	0.0243 (10)	
Na3	0.6667	0.3333	0.9477 (3)	0.0248 (6)	
O13	0.0000	0.0000	0.8007 (19)	0.041 (2)*	0.50
O14	0.3333	0.6667	0.7692 (9)	0.090 (3)*	

### Atomic displacement parameters (Å^2^)

**Table:**

	*U*^11^	*U*^22^	*U*^33^	*U*^12^	*U*^13^	*U*^23^
Mn2	0.0140 (2)	0.01171 (19)	0.0136 (2)	0.00596 (15)	−0.00045 (17)	0.00050 (14)
P1	0.0127 (4)	0.0114 (4)	0.0093 (4)	0.0061 (3)	0.0005 (3)	0.0014 (3)
P2	0.0128 (4)	0.0126 (4)	0.0103 (4)	0.0061 (4)	−0.0003 (4)	0.0009 (3)
B1	0.0110 (17)	0.0120 (17)	0.0113 (18)	0.0050 (14)	0.0000 (15)	0.0011 (15)
B2	0.018 (2)	0.0153 (18)	0.014 (2)	0.0096 (16)	0.0016 (16)	0.0004 (16)
B3	0.0135 (13)	0.0121 (12)	0.0206 (17)	0.0054 (10)	0.0010 (19)	0.0038 (19)
O1	0.0212 (13)	0.0161 (12)	0.0113 (14)	0.0079 (11)	0.0016 (11)	0.0013 (10)
O2	0.0210 (14)	0.0136 (12)	0.0125 (12)	0.0076 (11)	0.0022 (11)	0.0018 (10)
O3	0.0168 (13)	0.0148 (12)	0.0128 (14)	0.0075 (10)	−0.0020 (11)	0.0041 (10)
O4	0.0158 (12)	0.0161 (12)	0.0214 (14)	0.0081 (11)	0.0002 (11)	−0.0001 (11)
O5	0.0188 (13)	0.0134 (12)	0.0129 (12)	0.0078 (10)	0.0019 (11)	0.0022 (10)
O6	0.0211 (13)	0.0129 (12)	0.0184 (15)	0.0074 (11)	0.0021 (12)	0.0014 (11)
O7	0.0179 (14)	0.0180 (13)	0.0150 (15)	0.0061 (11)	−0.0019 (12)	0.0034 (11)
O8	0.0124 (11)	0.0162 (12)	0.0229 (14)	0.0064 (10)	0.0007 (11)	−0.0036 (11)
O9	0.0159 (12)	0.0160 (12)	0.0086 (12)	0.0053 (10)	0.0001 (11)	−0.0001 (11)
O10	0.0151 (8)	0.0132 (8)	0.0117 (8)	0.0074 (7)	−0.0023 (13)	−0.0018 (13)
O11	0.0146 (12)	0.0156 (12)	0.0142 (14)	0.0055 (10)	−0.0013 (11)	−0.0007 (11)
O12	0.0143 (10)	0.0297 (12)	0.0193 (13)	0.0055 (9)	−0.0020 (14)	0.0008 (15)
Na1	0.0289 (7)	0.0381 (7)	0.0212 (7)	0.0215 (6)	−0.0001 (10)	−0.0012 (11)
Na2	0.0238 (8)	0.0238 (8)	0.0278 (15)	0.0119 (4)	0.000	0.000
N	0.0161 (12)	0.0161 (12)	0.041 (3)	0.0081 (6)	0.000	0.000
Na3	0.0241 (8)	0.0241 (8)	0.0261 (14)	0.0121 (4)	0.000	0.000

### Geometric parameters (Å, °)

**Table:**

Mn2—O8	2.118 (3)	O5—B1^ix^	1.475 (5)
Mn2—O1^i^	2.126 (3)	O5—Na1^iv^	2.584 (3)
Mn2—O6	2.127 (3)	O6—Na1^iv^	2.434 (4)
Mn2—O4^ii^	2.162 (3)	O7—P2^vii^	1.562 (3)
Mn2—O9	2.303 (3)	O8—P2^x^	1.505 (3)
Mn2—O11^i^	2.326 (4)	O8—Na3^i^	2.376 (3)
Mn2—Na3^i^	3.6069 (13)	O9—B3^iv^	1.354 (6)
Mn2—Na2^i^	3.6582 (12)	O9—B1^iv^	1.493 (5)
P1—O1^i^	1.513 (3)	O10—Na1^iii^	2.360 (2)
P1—O4	1.514 (3)	O11—Mn2^vii^	2.326 (4)
P1—O2^i^	1.550 (3)	O12—Na1	2.656 (3)
P1—O3	1.555 (3)	O12—Na3	2.768 (4)
P1—Na2^i^	3.0544 (12)	O12—H1	0.86 (8)
P1—Na1^i^	3.094 (3)	Na1—O10^x^	2.360 (2)
P2—O6	1.500 (3)	Na1—O6^vi^	2.434 (4)
P2—O8^iii^	1.505 (3)	Na1—O5^vi^	2.584 (3)
P2—O5	1.562 (3)	Na1—P1^vii^	3.094 (3)
P2—O7^i^	1.562 (3)	Na1—P2^vi^	3.118 (3)
P2—Na1^iv^	3.118 (3)	Na1—H1	2.66 (8)
P2—Na3^i^	3.4270 (19)	Na2—O14	2.232 (11)
B1—O10	1.430 (5)	Na2—O4^xi^	2.370 (3)
B1—O5^v^	1.475 (5)	Na2—O4^vii^	2.370 (3)
B1—O3^vi^	1.491 (5)	Na2—O4^xii^	2.370 (3)
B1—O9^vi^	1.493 (5)	Na2—O1^viii^	2.817 (3)
B2—O10	1.416 (6)	Na2—O1^ii^	2.817 (3)
B2—O7	1.466 (6)	Na2—P1^xi^	3.0544 (12)
B2—O2^ii^	1.494 (5)	Na2—P1^vii^	3.0544 (12)
B2—O11	1.509 (5)	Na2—P1^xii^	3.0544 (12)
B3—O9^vi^	1.354 (6)	Na2—Mn2^xi^	3.6582 (12)
B3—O11	1.371 (6)	Na2—Mn2^xii^	3.6582 (12)
B3—O12	1.386 (4)	Na3—O8^xiii^	2.376 (3)
O1—P1^vii^	1.513 (3)	Na3—O8^xiv^	2.376 (3)
O1—Mn2^vii^	2.126 (3)	Na3—O8^vii^	2.376 (3)
O1—Na1	2.407 (4)	Na3—O12^iii^	2.768 (4)
O1—Na2	2.817 (3)	Na3—O12^x^	2.768 (4)
O2—B2^viii^	1.494 (5)	Na3—P2^xiv^	3.4270 (19)
O2—P1^vii^	1.550 (3)	Na3—P2^xiii^	3.4270 (19)
O2—Na1	2.614 (4)	Na3—P2^vii^	3.4270 (19)
O3—B1^iv^	1.491 (5)	Na3—Mn2^xiii^	3.6069 (13)
O4—Mn2^viii^	2.162 (3)	Na3—Mn2^xiv^	3.6069 (13)
O4—Na2^i^	2.370 (3)	Na3—Mn2^vii^	3.6069 (13)
			
O8—Mn2—O1^i^	95.34 (12)	B3^iv^—O9—Mn2	120.6 (2)
O8—Mn2—O6	89.88 (12)	B1^iv^—O9—Mn2	118.7 (2)
O1^i^—Mn2—O6	174.49 (15)	B2—O10—B1	118.2 (2)
O8—Mn2—O4^ii^	174.93 (18)	B2—O10—Na1^iii^	115.7 (3)
O1^i^—Mn2—O4^ii^	88.22 (11)	B1—O10—Na1^iii^	119.3 (3)
O6—Mn2—O4^ii^	86.46 (12)	B3—O11—B2	117.6 (3)
O8—Mn2—O9	86.00 (12)	B3—O11—Mn2^vii^	122.7 (2)
O1^i^—Mn2—O9	82.24 (11)	B2—O11—Mn2^vii^	116.6 (3)
O6—Mn2—O9	96.39 (14)	B3—O12—Na1	115.5 (2)
O4^ii^—Mn2—O9	90.91 (12)	B3—O12—Na3	94.0 (3)
O8—Mn2—O11^i^	93.89 (12)	Na1—O12—Na3	125.72 (15)
O1^i^—Mn2—O11^i^	97.79 (14)	B3—O12—H1	106 (5)
O6—Mn2—O11^i^	83.59 (12)	Na1—O12—H1	81 (5)
O4^ii^—Mn2—O11^i^	89.20 (12)	Na3—O12—H1	135 (5)
O9—Mn2—O11^i^	179.89 (14)	O10^x^—Na1—O1	125.56 (16)
O1^i^—P1—O4	113.43 (17)	O10^x^—Na1—O6^vi^	120.09 (16)
O1^i^—P1—O2^i^	105.09 (17)	O1—Na1—O6^vi^	108.15 (9)
O4—P1—O2^i^	112.12 (18)	O10^x^—Na1—O5^vi^	114.43 (12)
O1^i^—P1—O3	111.51 (18)	O1—Na1—O5^vi^	111.70 (11)
O4—P1—O3	109.4 (2)	O6^vi^—Na1—O5^vi^	57.80 (11)
O2^i^—P1—O3	104.87 (18)	O10^x^—Na1—O2	117.98 (12)
O6—P2—O8^iii^	114.34 (17)	O1—Na1—O2	57.76 (11)
O6—P2—O5	104.92 (18)	O6^vi^—Na1—O2	111.69 (11)
O8^iii^—P2—O5	112.87 (17)	O5^vi^—Na1—O2	67.76 (7)
O6—P2—O7^i^	110.57 (19)	O10^x^—Na1—O12	80.64 (8)
O8^iii^—P2—O7^i^	109.5 (2)	O1—Na1—O12	85.06 (12)
O5—P2—O7^i^	104.11 (17)	O6^vi^—Na1—O12	79.46 (12)
O10—B1—O5^v^	109.7 (3)	O5^vi^—Na1—O12	136.89 (14)
O10—B1—O3^vi^	108.1 (3)	O2—Na1—O12	142.78 (14)
O5^v^—B1—O3^vi^	108.4 (3)	O14—Na2—O4^xi^	120.17 (10)
O10—B1—O9^vi^	110.9 (3)	O14—Na2—O4^vii^	120.17 (10)
O5^v^—B1—O9^vi^	110.6 (3)	O4^xi^—Na2—O4^vii^	96.95 (13)
O3^vi^—B1—O9^vi^	109.0 (3)	O14—Na2—O4^xii^	120.17 (10)
O10—B2—O7	109.1 (4)	O4^xi^—Na2—O4^xii^	96.95 (13)
O10—B2—O2^ii^	109.2 (3)	O4^vii^—Na2—O4^xii^	96.95 (13)
O7—B2—O2^ii^	107.9 (3)	O14—Na2—O1^viii^	91.45 (9)
O10—B2—O11	111.2 (3)	O4^xi^—Na2—O1^viii^	57.64 (10)
O7—B2—O11	110.1 (4)	O4^vii^—Na2—O1^viii^	69.66 (9)
O2^ii^—B2—O11	109.2 (4)	O4^xii^—Na2—O1^viii^	147.64 (16)
O9^vi^—B3—O11	123.0 (3)	O14—Na2—O1^ii^	91.45 (9)
O9^vi^—B3—O12	120.0 (5)	O4^xi^—Na2—O1^ii^	69.66 (10)
O11—B3—O12	117.0 (5)	O4^vii^—Na2—O1^ii^	147.64 (16)
P1^vii^—O1—Mn2^vii^	126.55 (19)	O4^xii^—Na2—O1^ii^	57.64 (10)
P1^vii^—O1—Na1	101.82 (16)	O1^viii^—Na2—O1^ii^	119.937 (9)
Mn2^vii^—O1—Na1	121.96 (15)	O14—Na2—O1	91.45 (9)
P1^vii^—O1—Na2	83.98 (13)	O4^xi^—Na2—O1	147.64 (16)
Mn2^vii^—O1—Na2	94.45 (11)	O4^vii^—Na2—O1	57.64 (10)
Na1—O1—Na2	123.48 (15)	O4^xii^—Na2—O1	69.66 (9)
B2^viii^—O2—P1^vii^	135.3 (3)	O1^viii^—Na2—O1	119.937 (9)
B2^viii^—O2—Na1	132.2 (3)	O1^ii^—Na2—O1	119.937 (8)
P1^vii^—O2—Na1	92.40 (14)	O8^xiii^—Na3—O8^xiv^	93.15 (13)
B1^iv^—O3—P1	127.0 (3)	O8^xiii^—Na3—O8^vii^	93.15 (13)
P1—O4—Mn2^viii^	127.87 (17)	O8^xiv^—Na3—O8^vii^	93.15 (13)
P1—O4—Na2^i^	101.43 (16)	O8^xiii^—Na3—O12^iii^	120.52 (9)
Mn2^viii^—O4—Na2^i^	107.56 (13)	O8^xiv^—Na3—O12^iii^	78.10 (11)
B1^ix^—O5—P2	131.9 (3)	O8^vii^—Na3—O12^iii^	145.34 (9)
B1^ix^—O5—Na1^iv^	133.0 (2)	O8^xiii^—Na3—O12^x^	78.10 (10)
P2—O5—Na1^iv^	94.29 (14)	O8^xiv^—Na3—O12^x^	145.34 (9)
P2—O6—Mn2	125.0 (2)	O8^vii^—Na3—O12^x^	120.52 (9)
P2—O6—Na1^iv^	102.20 (17)	O12^iii^—Na3—O12^x^	77.89 (13)
Mn2—O6—Na1^iv^	128.53 (15)	O8^xiii^—Na3—O12	145.34 (9)
B2—O7—P2^vii^	127.6 (3)	O8^xiv^—Na3—O12	120.52 (9)
P2^x^—O8—Mn2	128.59 (17)	O8^vii^—Na3—O12	78.10 (10)
P2^x^—O8—Na3^i^	122.40 (16)	O12^iii^—Na3—O12	77.89 (13)
Mn2—O8—Na3^i^	106.59 (13)	O12^x^—Na3—O12	77.89 (13)
B3^iv^—O9—B1^iv^	119.0 (3)		

Symmetry codes: (i) *x*, *y*, *z*−1; (ii) −*x*+*y*, −*x*+1, *z*; (iii) −*y*+1, *x*−*y*, *z*; (iv) −*x*+1, −*y*+1, *z*−1/2; (v) *x*−*y*, *x*, *z*+1/2; (vi) −*x*+1, −*y*+1, *z*+1/2; (vii) *x*, *y*, *z*+1; (viii) −*y*+1, *x*−*y*+1, *z*; (ix) *y*, −*x*+*y*, *z*−1/2; (x) −*x*+*y*+1, −*x*+1, *z*; (xi) −*y*+1, *x*−*y*+1, *z*+1; (xii) −*x*+*y*, −*x*+1, *z*+1; (xiii) −*x*+*y*+1, −*x*+1, *z*+1; (xiv) −*y*+1, *x*−*y*, *z*+1.

### Hydrogen-bond geometry (Å, °)

**Table:**

*D*—H···*A*	*D*—H	H···*A*	*D*···*A*	*D*—H···*A*
O12—H1···O4^xv^	0.86 (7)	2.11 (7)	2.959 (3)	167 (3)
O13—···.O10^xvi^	.	.	2.8035 (11)	.
O13—···.N^xvii^	.	.	3.077 (16)	.
O14—···.O8^v^	.	.	3.284 (5)	.
O14—···.O6^v^	.	.	3.333 (3)	.
N—···.O13^i^	.	.	2.988 (16)	.
N—···.O3^xviii^	.	.	2.991 (3)	.
N—···.O7^i^	.	.	3.047 (3)	.

Symmetry codes: (xv) *x*−*y*+1, *x*, *z*+1/2; (xvi) −*x*+*y*, −*x*, *z*; (xvii) −*x*, −*y*, *z*+1/2; (v) *x*−*y*, *x*, *z*+1/2; (i) *x*, *y*, *z*−1; (xviii) *x*−1, *y*−1, *z*.
